# Anti-Melanogenic Effects of Ethanol Extracts of the Leaves and Roots of *Patrinia villosa* (Thunb.) Juss through Their Inhibition of CREB and Induction of ERK and Autophagy

**DOI:** 10.3390/molecules25225375

**Published:** 2020-11-17

**Authors:** Deok Jeong, Sang Hee Park, Min-Ha Kim, Sarah Lee, Yoon Kyung Cho, You Ah Kim, Byoung Jun Park, Jongsung Lee, Hakhee Kang, Jae Youl Cho

**Affiliations:** 1 Department of Integrative Biotechnology and Biomedical, Institute for Convergence at SKKU (BICS), Sungkyunkwan University, Suwon 16419, Korea; jd279601@gmail.com; 2 Department of Biocosmetics, Sungkyunkwan University, Suwon 16419, Korea; 84701@naver.com; 3 National Institute of Biological Resources, Environmental Research Complex, Incheon 22689, Korea; bistorta@korea.kr (M.-H.K.); lsr57@korea.kr (S.L.); yoonkyoung1@korea.kr (Y.K.C.); 4 Skin Science Research Institute, Kolmar Korea Co. Ltd., Chungcheongbuk-do 28116, Korea; ahyou2@kolmar.co.kr (Y.A.K.); a2001@kolmar.co.kr (B.J.P.)

**Keywords:** *Patrinia villosa* (Thunb.) Juss, autophagy, CREB, ERK, melanogenesis

## Abstract

*Patrinia villosa* (Thunb.) Juss is a traditional herb commonly used in East Asia including Korea, Japan, and China. It has been administered to reduce and treat inflammation in Donguibogam, Korea. The mechanism for its anti-inflammatory effects has already been reported. In this study, we confirmed the efficacy of *Patrinia villosa* (Thunb.) Juss ethanol extract (Pv-EE) for inducing autophagy and investigate its anti-melanogenic properties. Melanin secretion and content were investigated using cells from the melanoma cell line B16F10. Pv-EE inhibited melanin in melanogenesis induced by α-melanocyte-stimulating hormone (α-MSH). The mechanism of inhibition of Pv-EE was confirmed by suppressing the mRNA of microphthalmia-associated transcription factor (MITF), decreasing the phosphorylation level of CREB, and increasing the phosphorylation of ERK. Finally, it was confirmed that Pv-EE induces autophagy through the autophagy markers LC3B and p62, and that the anti-melanogenic effect of Pv-EE is inhibited by the autophagy inhibitor 3-methyl adenine (3-MA). These results suggest that Pv-EE may be used as a skin protectant due to its anti-melanin properties including autophagy.

## 1. Introduction

Autophagy refers to the process of self-degrading unnecessary proteins or organelles in cellular homeostasis in response to various factors [1]. Autophagy is closely related to human diseases such as cancer, infectious diseases, and diabetes, and is known to affect aging [2]. This role of autophagy has been reported to play a pivotal role in various physiological responses of skin cells. [3]. Through recent studies, many studies have been reported on the role of autophagy in the process of skin melanogenesis. For example, induction of autophagy inhibits melanin formation through melanosome degradation, whereas inhibition of autophagy increases melanin formation [4,5,6]. Autophagy is known to play a major role in both induction and inhibition of melanogenesis. In the case of LC3B, which is known as a major protein in autophagosome biogenesis, it regulates melanogenesis by inducing the activity of the CREB signaling pathway [7]. Deficiency of the WD repeat domain phosphoinositide-interacting protein 1, another major protein in autophagosome biogenesis, decreases MITF and tyrosinases mRNA [3]. Despite the central role of autophagy in melanogenesis, the regulatory mechanism is not clearly understood yet.

Α-Melanocyte-stimulating hormones (α-MSH) trigger cell signaling by reacting with MC1R, a specific receptor located on the cell surface of melanocytes [8]. Cells activated by the reaction of the ligand and receptor induce the activity of the cyclic adenosine monophosphate (cAMP) pathway and ultimately promote the transfer of the cAMP-response element binding protein (CREB) transcription factor into the nucleus [9]. CREB that has been moved into the nucleus binds to DNA and induces the transcription of microphthalmia-associated transcription factor (MITF), which is a major protein in melanogenesis and promotes melanin formation [10]. As such, activated MITF is phosphorylated by extracellular signal-regulated kinase (ERK) of MAPKs and protein degradation is induced, resulting in inhibition of melanin production [11].

*Patrinia villosa* is widely used in East Asia including China, Korea, and Japan. In Donguibogam, written by Jun Heo in 1613, it has been traditionally administered as a medicinal agent to relieve inflammation [12]. Biological studies using *Patrinia villosa* (Thunb.) Juss. Ethanol extract (Pv-EE) have shown that it has anti-inflammatory and anti-nociceptive properties [13,14] as well as anti-tumor effects. [15]. Many recent studies have shown that autophagy regulates melanin formation [16,17,18,19]. We conducted a study on plant extracts that regulate melanogenesis through autophagy [20] and confirmed that Pv-EE induces autophagy. In this paper, we analyzed the regulation of melanogenesis using the ability of Pv-EE to induce autophagy.

## 2. Results and Discussion

### 2.1. Anti-Melanogenesis Effects of Ethanol Extracts of Patrinia villosa Prepared fron Leaf (lPv-EE) and Root (rPv-EE) in α-Melanocyte Stimulating Hormone (α-MSH)-Treated B16F10 Cells

**Figure 1:**
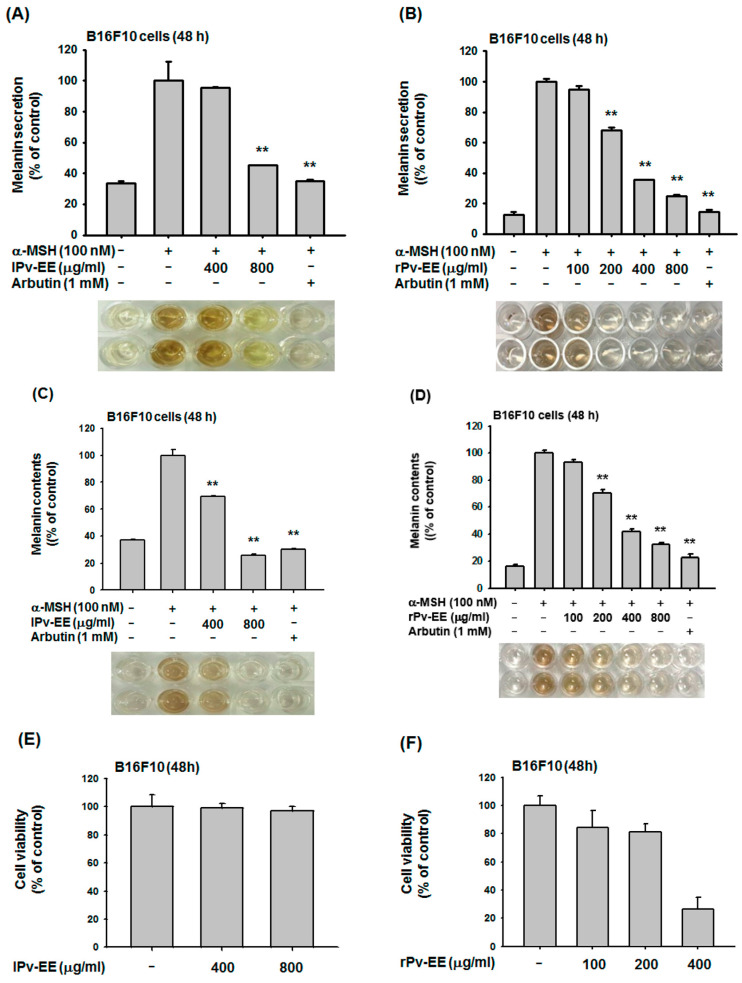
Anti-melanogenesis effects of ethanol extracts of *Patrinia villosa* prepared fron leaf (lPv-EE) and root (rPv-EE) in α-melanocyte stimulating hormone (α-MSH)-treated B16F10 Cells (**A**–**D**) The levels of melanin secretion and contents in B16F10 cells treated with -MSH (100 nM) in the presence or absence of lPv-EE (400 and 800 μg/mL), rPv-EE (100 to 800 μg/mL), or arbutin (1 mM) for 48 h. (**E**,**F**) Viability, determined using the MTT assay after 24 h, of B16F10 cells treated with various concentrations (400 and 800 μg/mL) of lPv-EE or (100 to 400 μg/mL) of rPv-EE. ** *p* < 0.01 compared to the control group.

To confirm the anti-melanogenic effects of Pv-EE from leaves and roots (lPv-EE and rPv-EE), we conducted a melanin formation assay using B16F10 cells. As a result, lPv-EE and rPv-EE clearly inhibited melanin secretion in a dose-dependent manner (Figure 1A,B). Melanin content analysis showed an inhibition effect of lPv-EE and rPv-EE in melanin formation (Figure 1C,D). To verify the cell viability of lPv-EE and rPv-EE, we performed an (3-4-5-Dimethylthiazol-2-yl)-2-5-diphenyltetrazolium bromide (MTT) assay using B16F10 cells. As shown in Figure 1E,F, lPv-EE was not cytotoxic at concentrations of 400–800 μg/mL. However, rPv-EE exhibited ~75% cytotoxicity at ~400 μg/mL, but at 100–200 μg/mL, rPV-EE decreased cell viability by only ~15–20%. Based on these data, we explored the inhibition effect of Pv-EE using 400–800 μg/mL of lPv-EE and 100–200 μg/mL of rPv-EE. These data indicated that Pv-EE has an excellent ability to regulate melanin.

### 2.2. Effect of lPv-EE and rPv-EE on MITF mRNA Expression Condition in α-MSH-Treated B16F10 Cells

**Figure 2:**
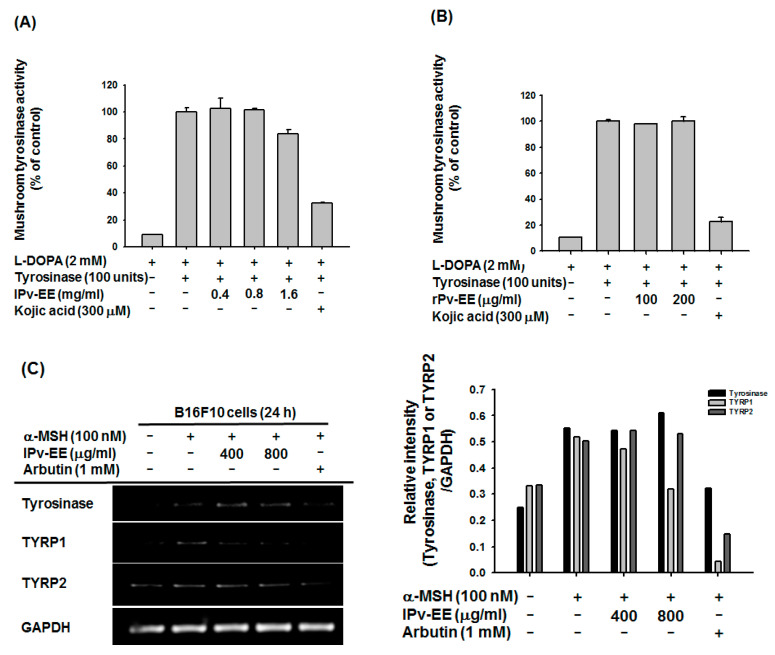

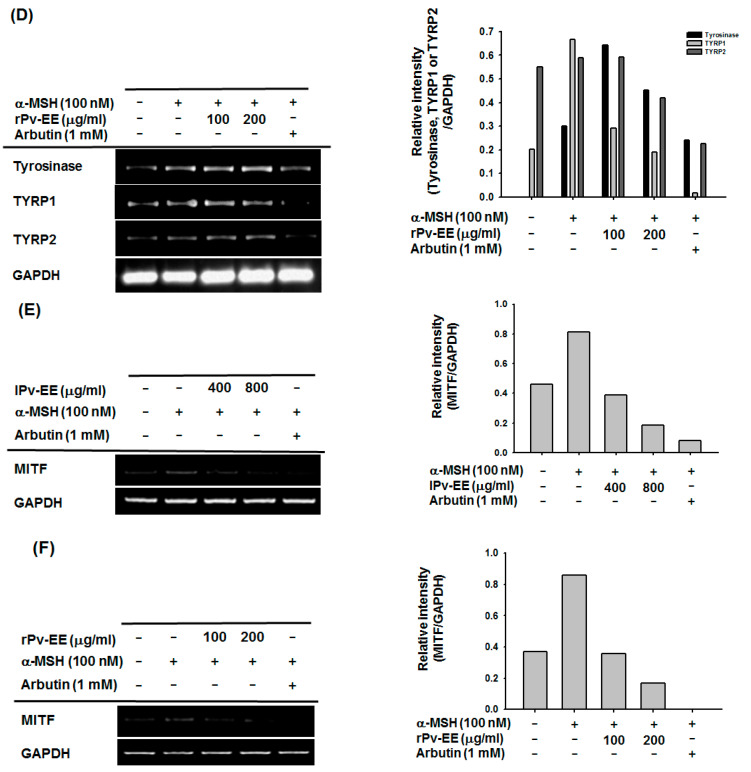
Effect of lPv-EE and rPv-EE on the MITF mRNA expression condition in α-MSH-treated B16F10 cells. (**A**,**B**) The effect of lPv-EE (400 to 1600 μg/mL), rPv-EE (100 and 200 μg/mL), or kojic acid (300 μM) on mushroom tyrosinase activity was determined by quantifying the activity of purified tyrosinase. (**C**–**F**) The mRNA levels, as determined by RT-PCR, of B16F10 cells treated with 100 nM α-MSH and lPv-EE (400 or 800 μg/mL) or rPv-EE (100 or 200 μg/mL) or 1 mM of arbutin for 24 h.

Next, we analyzed the signaling process of melanogenesis to explore the regulatory mechanism of Pv-EE. Tyrosinase is the key enzyme for melanin formation in melanocytes [21]. To demonstrate the anti-melanogenic effect of Pv-EE, we first measured the tyrosinase activity using mushroom tyrosinase and L-3,4-dihydroxyphenylalanine (L-DOPA). As shown in Figure 2A,B, neither lPv-EE nor rPv-EE regulated tyrosinase activity at the target concentration. As Pv-EE does not directly regulate tyrosinase activity, we confirmed mRNA expression of tyrosinase, TYRP1, and TYRP2. Interestingly, both types of Pv-EE regulated TYRP1, but not tyrosinase or TYRP2 (Figure 2C,D). The MITF mRNA expression, which is the transcription factor that regulates tyrosinase, TYRP1, and TYRP2, was decreased by both types of Pv-EE (Figure 2E,F). Thus, both types of Pv-EE inhibit melanogenesis by regulation of MITF expression.

### 2.3. Anti-Melanogenesis Mechanism of lPv-EE and rPv-EE in α-MSH-Treated B16F10 Cells

**Figure 3:**
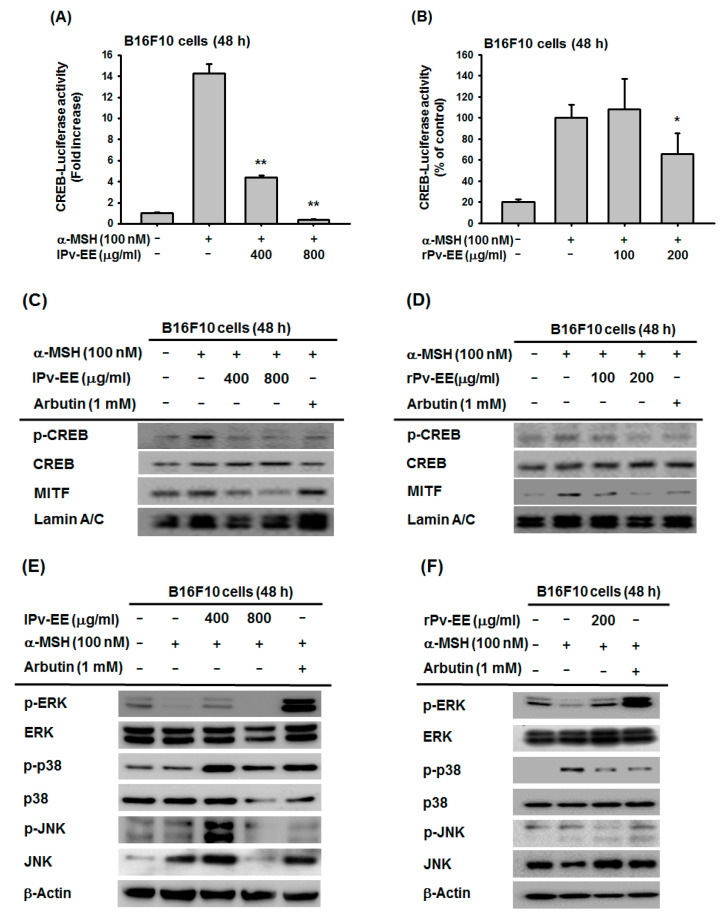
Anti-melanogenic mechanism of lPv-EE and rPv-EE in α-MSH-treated B16F10 cells. (**A**,**B**) The promoter binding activity of the transcription factor CREB was analyzed using a reporter gene assay. B16F10 cells were transfected with plasmids driving the expression of CREB-Luc (1 μg/mL) and β-gal (as a transfection control). After 24 h, some cells were treated with 100 nM α-MSH and lPv-EE (400 or 800 μg/mL) or rPv-EE (100 or 200 μg/mL) for 24 h. Luciferase activity was measured using a luminometer. (**C**–**F**) Levels of phosphorylated and total CREB, MITF, ERK, p38, JNK, and β-actin proteins were determined in B16F10 cells using phospho-specific or total antibodies for each protein. ** *p* < 0.01 compared to the normal group and * *p* < 0.05 compared to the control group.

α-MSH interacts with MC1R of melanocytes to activate the PKA-CREB signaling pathway, and it is well known that the MITF gene is expressed by activated CREB transcription factor [22]. Previous data showed that lPv-EE and rPv-EE strongly inhibit the MITF mRNA expression level on α-MSH-treated B16F10 cells. To clarify the inhibition mechanism of Pv-EE, we confirmed the PKA-CREB signaling pathway. CREB-luciferase activity was significantly decreased by 400–800 μg/mL lPv-EE and by 200 μg/mL rPv-EE (Figure 3A,B). Furthermore, we conducted the western blotting assay to confirm the molecular mechanism. As a result, the phospho-CREB protein and MITF total protein level were reduced by both types of Pv-EE (Figure 3C,D). These data showed that Pv-EE has an anti-melanogenic effect through the regulation of CREB activity.

It has been confirmed through many studies that the activity of MITF is regulated by MAPKs such as ERK, JNK, and p38 [23]. In the case of the B16F10 cell line, MAPKs are hyperactivated [24,25], and phospho-ERK, which inhibits MITF activity, decreases when induced by the α-MSH-mediated signaling pathway [20]. To prove the inhibition mechanism of both types of Pv-EE in the MAPKs pathway, we performed a western blotting assay to measure the amount of each protein. The expression of phospho-ERK increased at a concentration of 400 μg/mL of lPv-EE or 200 μg/mL of rPv-EE (Figure 3E,F). Based on these data, both lPv-EE and rPv-EE induce the activity of phospho-ERK to regulate melanogenesis.

### 2.4. Effect of Ethanol Extract of Patrinia villosa (Pc-EE) on Autophagy

**Figure 4:**
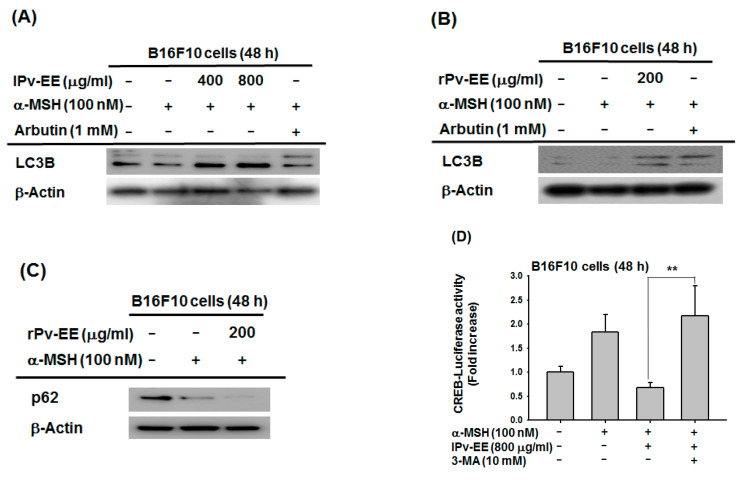

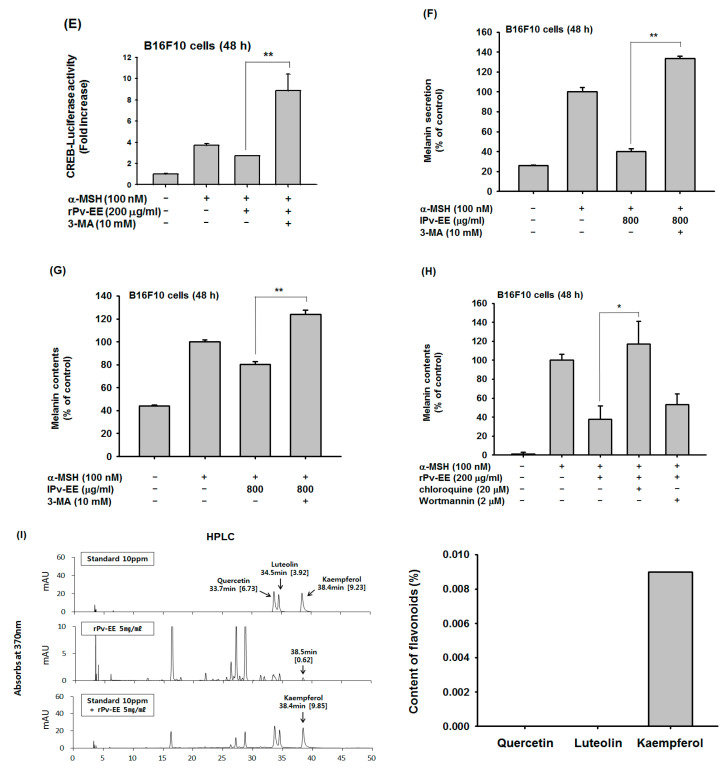
Effect of ethanol extract of *Patrinia villosa* (Pv-EE) on autophagy. (**A**–**C**) Levels of total LC3B, p62, and β-actin proteins were determined in B16F10 cells using the total antibodies for each protein. (**D**,**E**) The promoter binding activity of the transcription factor CREB was analyzed using a reporter gene assay. B16F10 cells were transfected with plasmids driving the expression of CREB-Luc (1 μg/mL) and β-gal (as a transfection control). After 24 h, some of the cells were treated with 100 nM α-MSH and lPv-EE (800 μg/mL) or rPv-EE (200 μg/mL) or 3-MA (10 mM) for 48 h. Luciferase activity was measured using a luminometer. (**F**–**H**) The levels of melanin secretion and contents in B16F10 cells treated with α-MSH (100 nM) in the presence or absence of lPv-EE (800 μg/mL) or 3-MA (10 mM) or chloroquine (20 μM) or Wortmannin (2 μM) for 48 h were then determined. (**I**) The phytochemical profile of rPv-EE was analyzed by HPLC using standard compounds (quercetin, luteolin, and kaempferol). ** *p* < 0.01 compared to the normal group and * *p* < 0.05 compared to the control group.

Recent studies indicated that autophagy plays a major role in melanogenesis [6,26], and our previous studies showed that the induction of autophagy inhibits melanogenesis [20]. To study the potential of Pv-EE to regulate autophagy, we measured LC3B or p62, a marker of autophagy, in α-MSH-induced B16F10 cells. As a result, both types of Pv-EE (400–800 μg/mL lPv-EE and 200 μg/mL rPv-EE) induced the LC3B protein level (Figure 4A,B). Moreover, when 200 μg/mL of rPv-EE was treated, another autophagy marker, p62, was decreased through increasing autophagy (Figure 4C). These data indicated that Pv-EE could regulate melanogenesis through the activation of autophagy. To support this hypothesis, we measured the CREB-luciferase activity by treatment with the autophagy inhibitor 3-methyladenine (3-MA). Surprisingly, CREB-luciferase activity that was decreased by both types of Pv-EE was recovered by 3-MA (Figure 4D,E). It is well known that the PKA-CREB signaling pathway degrades lipids by inducing autophagy-related genes such as Atg7, Ulk1, and Tfeb in nutrient-deprived conditions by recruiting the coactivator CRTC2 [27,28]. In contrast, another paper suggested that the activation of the PKA-CREB signaling pathway not only induced autophagy, but also makes feedback regulation of PKA-CREB by autophagy activity [29]. Our results showed that autophagic response increased by Pv-EE rather reduced CREB luciferase activity, while autophagy inhibitor 3-MA recovered its activity (Figure 4D–G). Therefore, it can be concluded that increased autophagy activity by Pv-EE could regulate the CREB signaling pathway through feedback regulation. Furthermore, we identified melanin secretion and content levels using autophagy inhibitors. As a result, melanin secretion and content levels were increased by 3-MA in the cells treated with 800 μg/mL of lPv-EE (Figure 4F,G). Class III PI3K is an important protein for lysosomal degradation signaling pathway [30]. 3-MA was reported to block autophagy through class III PI3K inhibition [31]. However, there was another report that 3-MA can specifically inhibit the activity of PI3K [32]. Based on this, there is a possibility that the role of Pv-EE may simply activate the PI3K-AKT signaling pathway, but not upregulate autophagy. To clarify this, we employed the lysosome inhibitor chloroquine and the PI3K-AKT inhibitor wortmannin to confirm their activity on melanin production. Interestingly, while melanin content was recovered by the lysosome inhibitor chloroquine, the PI3K-AKT inhibitor wortmannin did not show any abrogative effect (Figure 4H), implying that Pv-EE induces autophagic activity. These data show that Pv-EE has an anti-melanogenic effect via the induction of autophagy. Finally, we identified the phytochemical characteristics of rPv-EE using high-performance liquid chromatography (HPLC) with three flavonoid standards: quercetin, letolin, and kaempferol. The flavonoid of kaempferol was confirmed in rPv-EE at about 0.009% (Figure 4I).

## 3. Materials and Methods

### 3.1. Materials

The 95% ethanol extracts of the leaf and root of *Patrinia villosa* (Thunb.) Juss. (lPv-EE and rPV-EE, respectively) were obtained from the National Institute of Biological Resources (https://www.nibr.go.kr/), Incheon, Korea). (3-4-5-Dimethylthiazol-2-yl)-2-5-diphenyltetrazolium bromide (MTT), 5-hydroxy-2-hydroxymethyl-4H-pyranone (Kojic acid), monophenol monooxygenase (tyrosinase from mushroom), 4-hydroxyphenyl-β-D-glucopyranoside (arbutin), α-MSH, and L-DOPA ethyl ester were bought from Sigma Chemical Co. (St. Louis, MO, USA). The luciferase plasmids, which harbor promoter binding sites with CREB, were used as reported earlier [20]. TRIzol reagent was obtained from the Molecular Research Center, Inc. (Montgomery, OH, USA). Fetal bovine serum, Dulbecco’s modified Eagle’s media (DMEM), and phenol red-free DMEM were purchased from Gibco (Grand Island, NY, USA). B16F10 cells were received from ATCC (Rockville, MD, USA). All other chemicals were obtained from Sigma Chemical Co (St. Louis, MO, USA). Total and phospho-specific antibodies were purchased from Cell Signaling Technology (Beverly, MA, USA) as reported previously [33]. 

### 3.2. Cell Culture

B16F10 cells were cultured in DMEM containing phenol red that was supplemented with 10% fetal bovine serum and 1% antibiotics (penicillin and streptomycin). All cell lines were cultured in a CO_2_ incubator at 37 °C.

### 3.3. High-Performance Liquid Chromatography (HPLC)

The phytochemical characteristics of Pv-EE were analyzed with HPLC using the standard compounds quercetin, luteolin, and kaempferol. The HPLC was operated as described previously [20].

### 3.4. Cell Viability Assay

B16F10 cells were cultured in 24-well plates at a density of 5 × 10^4^ cells/well in fresh complete culture medium. Cells were treated with 400 and 800 μg/mL of lPv-EE or 100, 200, and 400 μg/mL of rPv-EE for each experimental condition. Cell viability was determined with a conventional MTT assay [20].

### 3.5. Melanin Formation Test

For the melanin formation assay, B16F10 cells (1 × 10^5^ cells/well in 12-well plates) were incubated for 24 h. After 24 h, the culture media were changed with fresh DMEM (phenol red-free) and treated with 100 nM of α-MSH, 400 and 800 μg/mL of lPv-EE, or 100, 200, 400, and 800 μg/mL of rPv-EE, 1 mM of arbutin, or 10 mM of 3-MA for 48 h. The melanin secretions and contents were analyzed using a protocol described previously [20].

### 3.6. Tyrosinase Assay

For the tyrosinase assay, 50 μL of 6 mM L-DOPA (dissolved in 50 mM of potassium phosphate buffer (pH 6.8)) were treated in 96-well plates. After treatment, 50 μL of the compound dissolved in potassium phosphate buffer (400, 800, and 1600 μg/mL of lPv-EE or 100 and 200 μg/mL of rPv-EE or 300 μM of kojic acid (dissolved in potassium phosphate buffer)) were added and incubated at room temperature for 15 min. After 15 min, mushroom tyrosinase (100 units/mL) dissolved in potassium phosphate buffer was then added to the mixture [34]. The absorbance of the mixture at 475 nm was then immediately measured using a multi-detection microplate reader.

### 3.7. Analysis of mRNA Levels by Reverse Transcriptase-Polymerase Chain Reaction (RT-PCR)

To quantify the levels of mRNA expression, B16F10 cells were treated with α-MSH (100 nM) together with lPv-EE (400 and 800 μg/mL) or rPv-EE (100 and 200 μg/mL). Total RNA was then isolated with the TRIzol reagent, according to the manufacturer’s instructions. RT-PCR was performed as described previously [35]. The primers used in this study are listed in Table 1.

### 3.8. Plasmid Transfection and Luciferase Reporter Gene Assay

For the luciferase reporter gene assay, B16F10 cells (5.0 × 10^4^ cells/well in 24-well plates) were transfected with 0.8 μg/mL of plasmids driving the expression of β-galactosidase and CREB-luciferase reporter gene. Transfection used the polyethyleneimine method as reported previously [34]. B16F10 cells were incubated for 24 h. After 24 h, the cells were treated with 400 or 800 μg/mL of lPv-EE or 100 or 200 μg/mL of rPv-EE or 10 mM of 3-MA for an additional 48 h. CREB-luciferase was induced by 100 nM of α-MSH for 48 h.

### 3.9. Immunoblotting

Total lysates prepared from the B16F10 cells were subjected to the western blotting assay of the total and phospho-forms of tyrosinase, CREB, MITF, Lamic A/C, JNK, ERK, p38, LC3B, and β-actin. Immunoreactive bands were visualized as described previously [20,36].

### 3.10. Statistical Analysis

All data are presented as means ± standard deviation and each experiment consisted of three or four replications. The Mann–Whitney U test was used to analyze the statistical difference between groups. A *p* value < 0.05 was regarded as statistically significant. All statistical tests were performed using SPSS software (version 22.0, 2013; IBM Corp., Armonk, NY, USA).

## 4. Conclusions

**Figure 5:**
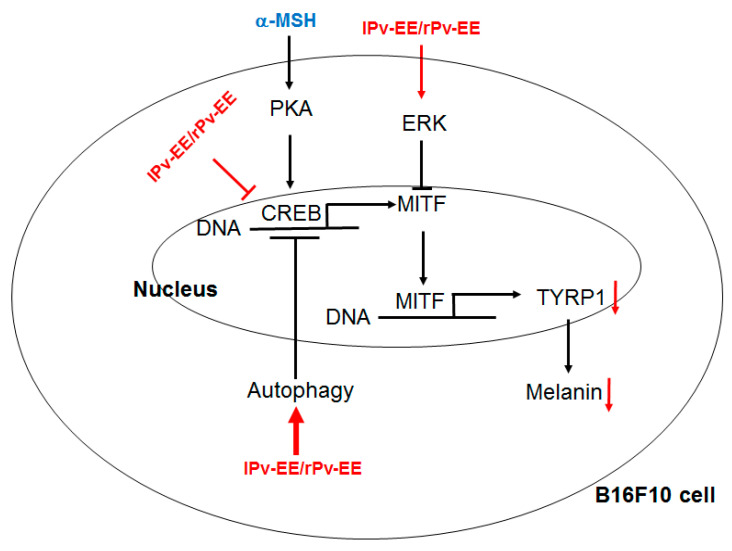
Putative inhibitory pathway of lPv-EE and rPv-EE.

In summary, we demonstrated that Pv-EE has an excellent anti-melanogenic effect on α-MSH-induced melanogenesis. We also used molecular mechanism studies to show that Pv-EE induces autophagy and regulates CREB and MAPKs signaling processes and also exhibits anti-melanin in melanocytes (Figure 5). Although the activity of LC3B, one of the proteins of autophagosome biogenesis, induces CREB activity and promotes melanin production [7], our results suggest that Pv-EE can downregulate melanin production through an increase in autophagy. The recovery of CREB luciferase activity by autophagy inhibition (Figure 4D,E) shows that autophagy increased by Pv-EE could modulate the CREB-signaling pathway by induction of negative feedback loop regulation [29]. Since there are some papers that state that autophagy may negatively or positively affect the cAMP-PKA-AMPK-SIRT1 signaling pathway including feedback regulation between PKA and autophagy [37,38], we will further examine the detailed mechanism to explain the role of autophagy in anti-melanogenic responses. 

Finally, kaempferol was detected in the analysis of Pv-EE’s phytochemical properties (Figure 4G). Kaempferol has been previously reported to have anti-melanogenesis [39] and autophagy-inducing effects [40]. Therefore, kaempferol appears to play an important role in the efficacy of Pv-EE in this research. Thus, this study shows that Pv-EE inhibits melanin formation, and therefore could potentially be used in drugs or cosmetics as a skin protectant due to its anti-melanin properties including autophagy.

## Figures and Tables

**Table 1:** Polymerase chain reaction (PCR) primers used in this study.

Name		Sequence (5′ to 3′)
*Mouse*		
Tyrosinase	F	GTCCACTCACAGGGATAGCAG
	R	AGAGTCTCTGTTATGGCCGA
TYRP1	F	ATGGAACGGGAGGACAAACC
	R	TCCTGACCTGGCCATTGAAC
TYRP2	F	CAGTTTCCCCGAGTCTGCAT
	R	GTCTAAGGCGCCCAAGAACT
MITF	F	GGGAGCTCACAGCGTGTATT
	R	CTAGCCTGCATCTCCAGCTC
GAPDH	F	ACCACAGTCCATGCCATCAC
	R	CCACCACCCTGTTGCTGTAG
